# Reflections on the Asan Medical Center Plastic Surgery Visit: A Well-Organized Specialized Team and Excellent Leadership

**DOI:** 10.1055/a-2556-0673

**Published:** 2025-05-15

**Authors:** Hatan Mortada

**Affiliations:** 1 Division of Plastic Surgery, Department of Surgery, King Saud University Medical City, King Saud University, Riyadh, Saudi Arabia; 2 Department of Plastic Surgery and Burn Unit, King Saud Medical City, Riyadh, Saudi Arabia

**Keywords:** plastic surgery, reconstructive microsurgery, fellowship, Asan Medical Center, surgical education

## Abstract

Asan Medical Center in Seoul, South Korea, is renowned for its excellence in plastic and reconstructive surgery. This paper aims to share insights from a 2-week fellowship experience in the Department of Plastic Surgery, led by Professor Jong Woo Choi. The program offers comprehensive clinical observership, including participation in daily conferences, ward rounds, outpatient clinics, and surgical procedures. Observers gain exposure to complex microsurgical cases, benefiting from the department's high case volume and state-of-the-art facilities. The center's commitment to pioneering research and innovation provides exposure to cutting-edge techniques and fosters collaboration. Personal reflections highlight the program's impact on professional development and the supportive learning environment created by Professor Hong and his team. This fellowship offers an unparalleled opportunity for surgeons to enhance their expertise in reconstructive microsurgery, engage with leading experts, and witness advanced patient care. The experience provides valuable insights and inspiration for those considering similar educational pursuits in plastic and reconstructive surgery.

Asan Medical Center (AMC), situated in Seoul, South Korea, is a world-renowned institution excelling in various medical specialties, particularly in plastic and reconstructive surgery. As one of the largest hospitals in Korea, with over 2,700 beds, it serves a population of over 9.7 million residents in the Seoul metropolitan area. 1 This article shares insights from my enriching 2-week observership at AMC, one of the highest-volume centers for plastic and reconstructive surgery. The short duration of this observership provided a concentrated exposure to advanced techniques and high-volume practice, offering a unique perspective on the institution's approach to complex microsurgical cases.

## Department Structure and Dynamics

The Department of Plastic Surgery at AMC is currently chaired by Professor Jong Woo Choi, with Professor Joon Pio Hong serving as the former chair. The department is organized into three primary teams:

1. Extremity Reconstruction and Lymphedema Team (Team A).
2. Facial Deformity and Craniofacial Reconstruction Team (Team C).
3. Breast Reconstruction Team (Team B).

This structure allows for specialized focus and expertise within each area of plastic and reconstructive surgery.

## Clinical Observership and Hands-on Experience

AMC stands out for its impressive volume of complex cases in plastic and reconstructive surgery. AMC's Plastic Surgery Department performs over 4,500 surgical cases annually, with approximately 30 SCI journal publications each year. 2 This high-volume environment provides an unparalleled learning opportunity for visiting surgeons and trainees. During my 2-week observership, I had the privilege of immersing myself in the department's daily activities:

- Attending morning conferences and ward rounds.
- Observing outpatient clinics.
- Witnessing numerous surgical procedures in state-of-the-art operating theaters.

**Fig. 1:**
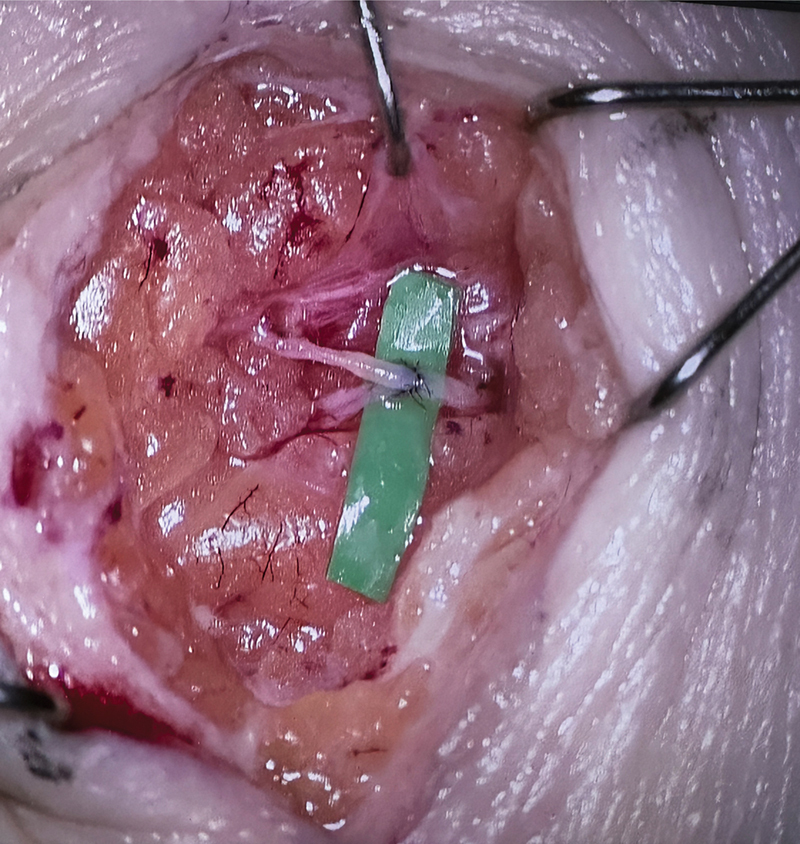
A side-to-end lymphovenous anastomosis being performed at the Asan Medical Center for a patient with four-limb congenital lymphedema.

Under the guidance of Professor Hong and his skilled team, I gained exposure to a wide range of advanced microsurgical procedures. A particularly memorable case was a side-to-end lymphovenous anastomosis for a patient with four-limb congenital lymphedema, highlighting the team's expertise in complex reconstructive challenges ( Fig. 1 ).

## Key Learnings and Their Impact

During my observership at the AMC, I gained insights into advanced surgical techniques, high-volume practice efficiency, multidisciplinary collaboration, innovative research integration, patient-centered care in a high-tech environment, and the importance of continuous learning. These observations and lessons are summarized in Table 1 .

**Table 1:** Summary of observations and lessons learned during the observership at the Asan Medical Center

Key area	Observation	Lesson learned
Advanced microsurgical techniques	Innovative approaches to lymphovenous anastomosis for complex lymphedema cases	Refined techniques for vessel preparation and anastomosis in confined spaces
Multidisciplinary collaboration	Regular tumor board meetings integrating various specialties	Importance of early plastic surgery involvement in oncological cases
Innovative research integration	Rapid translation of research into clinical practice	Techniques for staying current with literature and applying innovations
Continuous learning culture	Daily case presentations and literature reviews	Fostering a culture of ongoing education within the surgical team
Team humility and camaraderie	Observed the humility and teamwork of the surgical team	Learned the importance of maintaining humility and fostering a collaborative environment
Use of innovative techniques	Utilization of ultrasound in microsurgical procedures	Gained insights into the benefits of integrating ultrasound for precision in surgery
Commitment to teaching	Noted the team's dedication to teaching and mentoring	Inspired to prioritize teaching and knowledge sharing

## Training Opportunities at the Asan Medical Center

AMC offers various training opportunities for plastic surgeons at different career stages, including but not limited to Plastic Surgery Residency, Microsurgery Fellowships, Craniofacial Fellowships, Breast Reconstruction Fellowships, and Short-term Observerships. The 2-week observership I experienced is ideal for practicing surgeons or residents seeking focused training in specific techniques or subspecialties. Participants can attend daily rounds, observe surgeries, engage in discussions, and gain insights into ongoing research and innovations. To apply, surgeons should submit their CV, a letter of intent, and a recommendation letter from their department chair or supervisor to the AMC's International Healthcare Center.

## Personal Reflections and Gratitude

**Fig. 2:**
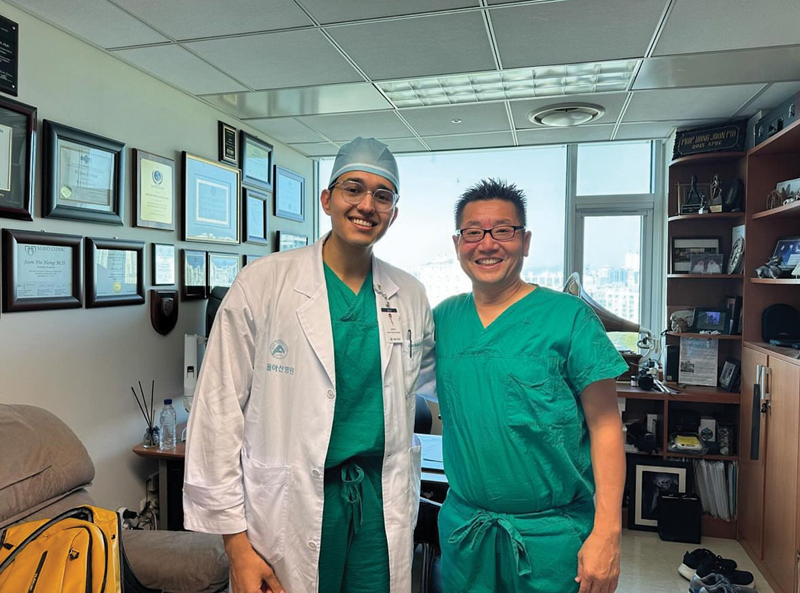
The author and Professor Joon Pio Hong after their meeting, discussing the author's goals and aspirations, a practice Professor Hong upholds with all his visitors.

My 2-week visit to AMC was an enriching experience that significantly exceeded my expectations. I am grateful to Professor Joon Pio Hong, Professor Changsik John Pak, Professor Hyunsuk Peter Suh, and the entire team for their warm welcome and commitment to sharing their knowledge and expertise. The skills and insights I gained during this visit will have a lasting impact on my career as a plastic surgeon. One aspect that particularly touched me was Professor Hong's genuine interest in his visitors' goals and aspirations. He made it a point to sit down with each of us, discuss our future plans, and offer valuable advice and encouragement ( Fig. 2 ). This personal touch exemplifies his commitment to fostering the growth of young surgeons and creating a supportive learning environment.

## Conclusion

The observership at AMC has profoundly influenced my approach to plastic and reconstructive surgery. The exposure to advanced techniques, innovative research, and efficient high-volume practice has enhanced my surgical skills and broadened my perspective on patient care. I strongly recommend this program to any surgeon seeking to enhance their expertise in reconstructive microsurgery. The skills and insights I gained during this observership will undoubtedly shape my career as a plastic surgeon. I unreservedly endorse this program for any surgeon aiming to advance their skills in reconstructive microsurgery. The experience at AMC stands as a testament to the value of international exchange in medical education and the continuous pursuit of excellence in our field.
